# It Depends Who Is Watching You: 3-D Agent Cues Increase Fairness

**DOI:** 10.1371/journal.pone.0148845

**Published:** 2016-02-09

**Authors:** Jan Krátký, John J. McGraw, Dimitris Xygalatas, Panagiotis Mitkidis, Paul Reddish

**Affiliations:** 1 Laboratory for the Experimental Research of Religion, Masaryk University, Brno, Czech Republic; 2 Interacting Minds Centre, Department of Culture and Society, Aarhus University, Aarhus, Denmark; 3 Departments of Religious Studies and Central American Studies, California State University Northridge, Northridge, California, United States of America; 4 Department of Anthropology, University of Connecticut, Storrs, Connecticut, United States of America; 5 Center for Advanced Hindsight, Social Science Research Institute, Duke University, Durham, North Carolina, United States of America; 6 Interdisciplinary Center for Organizational Architecture, Department of Management, School of Business and Social Sciences, Aarhus University, Aarhus, Denmark; Ghent University, BELGIUM

## Abstract

Laboratory and field studies have demonstrated that exposure to cues of intentional agents in the form of eyes can increase prosocial behavior. However, previous research mostly used 2-dimensional depictions as experimental stimuli. Thus far no study has examined the influence of the spatial properties of agency cues on this prosocial effect. To investigate the role of dimensionality of agency cues on fairness, 345 participants engaged in a decision-making task in a naturalistic setting. The experimental treatment included a 3-dimensional pseudo-realistic model of a human head and a 2-dimensional picture of the same object. The control stimuli consisted of a real plant and its 2-D image. Our results partly support the findings of previous studies that cues of intentional agents increase prosocial behavior. However, this effect was only found for the 3-D cues, suggesting that dimensionality is a critical variable in triggering these effects in a real-world settings. Our research sheds light on a hitherto unexplored aspect of the effects of environmental cues and their morphological properties on decision-making.

## Introduction

Reputational models of human behavior predict that uncooperative individuals risk being excluded from possible future interactions [1,2] or otherwise incur costs imposed by others [3,4], suggesting that cooperative behavior may be fitness-enhancing. Thus, being monitored raises reputational concerns and consequently leads to the preference of socially desirable cooperative strategies. Since mechanisms guiding the intention to cooperate have been developed under the pressure of reputational concerns and the possible threat of punishment they are highly sensitive to real or putative signals implying an awareness, at some level, that one’s actions are being monitored [5]. Interestingly, perceived monitoring by social others can be elicited by the presence of artificial signals that merely cue agency by way of a strong resemblance to human beings [6,7]. Such cues affect our behavioral responses in subtle, probably unconscious ways [8,9]. The present study investigates the morphological features of material objects (e.g., human faces) and whether they lead to improved levels of fairness by eliciting the sense of being watched

Due to the evolutionary significance of agency detection, the propensity to detect intentional agents on the basis of subtle morphological features exists even among lower vertebrates, and seems to have gradually developed throughout evolutionary history [10–13]. Humans, in particular, are prone to over-detecting agency and modifying their behaviour accordingly [12,14–17]. The detection of such cues triggers the attribution of mental states (i.e. feelings, desires and intentions) and promotes the tendency to explain occurring events in a prototypically mentalistic manner [18,19]. Although our propensity to anthropomorphize may lead to frequent misattributions [20–22], the ability to perceive others as intentional and sentient beings [23] is an essential component of social and more moral cognition. It is a prerequisite for the detection of moral transgressions [24–27], and also underlies judgement of our own deeds [28].

Material structures that share morphological features with human agents suggest the presence of conspecifics [29,30]. When spotted, these material cues trigger specific types of socially meaningful behavioral responses [31]. This effect has been demonstrated in a number of studies that reported behavioral changes as well as greater awareness of one’s own socially sensitive behaviours after being exposed to images of agents or even just a salient feature, such as eyes. Among the first to document this effect in laboratory settings were Haley and Fessler [6], who found that participants primed by auditory and visual cues of human agents acted more prosocially in a dictator game. Similar behavioral responses to being watched or monitored were confirmed by researchers using economic games [32–34]. Even very subtle agency cues such as three dots in a “watching-eyes configuration” have been found to increase prosocial behavior [35]. This line of research has been extended by studies conducted in naturalistic settings which have found convergent evidence for the effects of image-based agency cues on socially desirable behaviours such as contribution to charitable causes [36,37]; time investment in public goods [38]; and reduced littering in public spaces [5,39]. Another study [40] used complex material environments such as a Hindu temple populated by sculptures and imagery of the deities as a experimental manipulation. Participants in that setting behaved in more cooperative ways than participants in a control setting and were more likely to invoke a sense of fairness as an explanation for their decisions. Similar evidence has also been documented by Ahmed and colleagues [41]. The present study was designed to test the effects of agency cues on fairness [42–44] in a real-world decision-making task. As noted by Rabin [45], the mutual expectations of cooperating parties occupy a key position when assessing whether a behaviour is fair. In our task, participants were offered a freely accessible product in exchange for their monetary contribution. However, the contribution was voluntary, entirely depending on each participant’s decision in response to a call for a fair contribution in the instructions.

In an earlier study, Bateson and her colleagues [7] manipulated their psychology department’s kitchenette and used faculty members as subjects, who were unaware of the true nature of the situation. The experimental stimuli consisted of photorealistic depictions of eyes, alternated on a weekly basis with control stimuli, which consisted of pictures of flowers. The stimuli were featured alongside instructions asking department members to contribute a specific amount of money in exchange for a hot beverage on an honorary system. The experimenters then recorded the total amount of monetary contributions added to a common pool honesty box and the amount of milk consumed as a measure of total beverage consumption. The results showed significantly higher monetary contributions to the common pool after exposure to pictures of eyes.

The study by Bateson and colleagues was constrained by a number of limitations. Participants came from a very limited subject pool consisting of only 48 department members, moreover it is unknown how many of them actually took part in the experiment. Additionally, of all the beverages consumed in the kitchenette (i.e. tea, coffee, milk), only the amount of milk was recorded and used as an index of consumption, thus not allowing for accurate measures regarding the two other beverages. Finally, there was no individual record of either contribution or consumption, hence it is possible that a few or even a single outlier may have caused the observed effect. The present study aimed to control for all the aforementioned factors by using a much larger sample (N = 345); by recording individual contributions; by limiting the offered products to a single beverage; and by controlling for abuse (e.g. more than one drink per person taken). These factors were managed while still maintaining the ecological validity of the study in a naturalistic setting.

Crucially, we introduced dimensionality of the stimuli as a variable to examine the effects of their spatial properties. Previous studies have generally primed participants with minimal cues of observation, such as photographs or schematic images of eyes only [6,7,36–39,46], as such features are arguably the most suggestive of the presence of an intentional agent. In our study, we used a sculpture of a human head that shared additional morphological elements with real human agents to further increase the salience of the experimental stimulus. This higher activation of agency detection, in turn, ought to trigger stronger responses and thus further amplify prosocial behavior.

Based on previous findings [6], our first hypothesis was that monetary contributions would be higher in the presence of an agency cue compared to control stimuli. Secondarily, we investigated whether dimensionality would moderate the effect of agency on monetary contributions. To assess these hypotheses, we conducted a quasi-experimental study in a naturalistic setting by manipulating the presence and dimensionality of experimental primes and examining their effects on a monetary contribution task.

## Methods

We used a 2x2 between-subjects factorial design. Our independent variables were agency (presence vs. absence of agentive characteristics in the presented stimuli) and dimensionality (2-dimensional vs. 3-dimensional stimuli).

### Participants

345 individuals (128 females) took part in the study. Our sample consisted of visitors to the main library of the Faculty of Science at Masaryk University in Brno, Czech Republic. Participants were self-distributed between the experimental conditions in the following way: 3D head (N = 106); 2D head (N = 83); 3D plant (N = 93); and 2D plant (N = 63).

### Stimuli

The manipulation involved varying the particular stimuli that were present in the experimental setting (see S1 Appendix. Experimental Stimuli). The 3D socially salient object was a pseudo-realistic three-dimensional sculpture of a human head (3D head) measuring 135mm (width) x 225mm (height) x 150 (depth). In the 3D socially non-salient condition, we used a plant (*Aloe brevifolia*) (3D Plant) measuring 270mm (height) x 100mm (width) x 100m (depth). In the 2-dimensional (2D) conditions, photographs of the aforementioned 3D objects were taken (background removed) and printed in color on A4 paper (2D Head and 2D Plant). 2D objects were of the same height and width as the 3D stimuli.

### Design and procedure

Participants were given the opportunity to get an energy drink and make an optional contribution to an “honesty box.” Drinks were placed in a small refrigerator with a transparent glass door, situated in the entrance hall of the library, a place that was highly frequented. The refrigerator was stocked with 20 cans of Red Bull which was replenished daily. Each day the experimental site was set up prior to the opening of the library at 9:00am and remained in place until either the stock was exhausted or three hours had transpired. Apart from these times, all objects and signs pertaining to our study were withdrawn from the site. The study was conducted over 20 consecutive weekdays with five days for each of the conditions. All four experimental conditions were randomized throughout all 20 experimental days.

A cover story was presented using a sign that informed visitors that the library was planning to open a canteen and needed to conduct simple market research to determine demand and appropriate prices for certain beverages. Visitors were encouraged to take one drink per person and contribute as much as they considered fair by placing the money in an envelope and dropping it into the honesty box (a transparent plastic box 560mm wide, 280mm high and 390mm deep). All contributions were made in Czech Koruna (CZK; 1 Koruna equalled approximately US$0.05 at the time of this study). At the beginning of each day, some envelopes were placed in the box to suggest to prospective participants that previous donations had taken place. Envelopes were numbered and marked for gender by a circular sign (for females) and a rectangular sign (for males) in two separate piles, allowing us to determine the order and gender of individual participants. The instructions were placed on a notice board, typeset in 24 point Times New Roman and printed on A3 paper, placed approximately 40 cm behind the honesty box. Participants were thus oblivious to the real purpose (and existence) of the study while being well-informed about the task and procedure.

The 3-dimensional stimuli were placed on a wooden pedestal (134 cm in height) in front of the notice board where the printed instructions appeared (see S2 Appendix. Experimental set-up). The 2D stimuli appeared on a printed poster that was placed on the notice board directly behind the position where the 3D stimuli would otherwise be exhibited. We ensured that the stimuli were presented in precisely the same position throughout the duration of the experiment, that is, even though 2D stimuli had different spatial characteristics than 3D stimuli, they were placed in the exact position and faced the same direction as the 3D stimuli. The wooden pedestal remained in place at all times. We ensured that there were no images of faces or any other photos suggesting a human or animal presence on the notice board or in the surrounding area that might compete for attention or otherwise interfere with the experimental stimuli. Informative posters and flyers, furniture, as well as objects of decorative use in the hall were kept in their original place throughout the course of the study as long as they did not exhibit any formal characteristics conveying presence of other agents

The entrance hall of the library that hosted our experiment was approximately 35 square meters (376ft^2^) in size and served as gateway to the library. The visitor traffic from which our participants were recruited varied over time. Visitors engaged in a variety of activities in the entrance hall, however these activities did not in any way interfere with the aim of our study. The setup was monitored by an experimenter in the next room via the library’s closed circuit camera that was accessed with the permission of library officials. This allowed the experimenter to check for possible abuse, ensure that gender distribution was not misreported, that there were no repeat visitors, and observe whether individuals took multiple drinks at a time or paid without taking a drink. Our observations indicated that none of the above was the case. Written approval for this study was obtained from the ethical review board of the Czech Association for the Study of Religions. In addition, we obtained written approval from the library to use the space and have access to the closed circuit camera footage, which was only available to the researchers as a live stream and was not recorded in any form.

## Results

Overall, there was a floor effect, with 149 participants (43.18% of all study participants) contributing no money (percentage per condition: 3D Head = 38.68%; 2D Head = 42.17%; 3D Plant = 44.09%; 2D Plant = 50.71%),

The high proportion of zero monetary contributions led us to initially examine whether our manipulation influenced participants’ tendency to make a contribution *or not*. We conducted a logistic regression with contributing (1) or not (0) dummy coded as the dependent variable and the factors of agency cue and dimensionality as predictors. This analysis revealed no statistically significant main effects or interactions (see Table 1).

**Table 1:** Estimates and SE from logistic regression model. Table shows that dimension and agency used as predictors of logistic regression model do not predict likelihood of monetary contributions. Note: R^2^ = .007 (Cox & Snell), .009 (Nagelkerke). Model χ^2^ (3) = 2.42, p = 0.49.

	B	SE	Wald	df	*p*
Agency	.348	.336	1.070	1	.301
Dimensionality	.269	.327	.678	1	.410
Agency x Dimensionality	-.124	.443	.079	1	.78

We next examined whether our manipulation influenced the amount participants gave. To this end, we first removed those participants with zero donations and further analyses were conducted only with those who made contributions (see Table 2).

**Table 2:** Mean monetary contributions. Table shows mean monetary contributions in conditions after removing zero contributions (in CZK).

	Dimensionality						
	3D				2D		
Cue of social presence	n	*M* (*SD*)	± 1SE		n	*M* (*SD*)	± 1SE
Head (presence)	65	13.18 (7.76)	.96		48	10.21 (5.47)	.79
Plant (absence)	52	9.81 (6.51)	.90		31	10.90 (6.90)	1.24

Skewness (1.27, *SE* = .33) and kurtosis (1.61, *SE* = .65) were significantly non-normal for the 3D Plant condition (*p*s < .05), but not so for the other three conditions. In order to compensate for the non-normality of our data we used bias-corrected accelerated bootstrapped data (CI = 95%, 10,000 samples) in subsequent analyses.

A 2x2 (agency vs. dimensionality) univariate factorial analysis of variance indicated a significant interaction of agency and dimensionality factors: *F*(1,192) = 4.11, *p* = .044. There were no significant main effects of agency, *F*(1,192) = 1.78, *p* = .184, and dimensionality, *F*(1,192) = 0.88, *p* = .351.

A simple effect analysis revealed a significant difference in monetary distribution between the 3D Head and 3D Plant conditions, *p* = .013. However, there was no difference in monetary contributions between 2D Head and 2D Plant (*p* = .630). Examining the simple effects of dimensionality, 3D Head produced significantly greater monetary contributions than 2D Head, *p* = .019, but no difference in dimensionality was found between 3D Plant and 2D Plant, *p* = .468.

To check the robustness of these analyses on non-normal data, we repeated these pairwise comparisons with non-parametric statistics. Mann-Whitney tests also showed a significant difference between the 3D Head and 3D Plant (Mann-Whitney *U* = 1258.5, *p* = .016) but the difference between the 3D Head and 2D Head was only marginally significant (Mann-Whitney *U* = 1269, *p* = .084). As with the bootstrapped comparisons above, the other two pairwise comparisons were not significant (2D Head vs. 2D Plant: Mann-Whitney *U* = 709.5, *p* = .726; 3D Plant vs. 2D Plant: Mann-Whitney *U* = 712.5, *p* = .378).

## Discussion

Earlier studies that explored the effect of agency cues on prosocial behavior typically used either lab-based experiments with formalized economic games as their dependent measures [6,35,47–49] or field experiments with only limited control [7]. The current study employed a real-world setting combining both ecological validity and a high level of control.

Our results provide only partial support for the first hypothesis, that the presence of agency stimuli would increase fairness, however support our second hypothesis, that 3-dimensional agency stimuli (3D head) would increase fairness compared to the 2-dimensional ones (2D head).

Analyzing the data, we first explored whether our manipulations increased the probability of a contribution, as suggested by the proportion between zero and non-zero contributions. We found that participants were not more likely to contribute something rather than nothing when exposed to socially salient cues. Interestingly, this result is inconsistent with the findings of a meta-analysis by Nettle at al. [50] who reported that cues of being watched increased the probability of donating something rather than nothing more than they influenced the donation’s mean. First, we hazard that this may be attributed to the nature of our experimental task which differed considerably from the lab-based experiments using economic games that Nettle and colleagues [50] included in their meta-analysis. Second, the instructions in our study the encouraged participants to make a monetary contribution of whatever amount they considered to be fair. e speculate that participants’ decisions that led to zero contributions were, in fact, not the real contributions but instead a sign of a rejection of the proposed situational frame and only true contributors were possibly those affected by our experimental stimuli. There might be two distinct cognitive mechanisms at play here–one driving the decision to contribute or not, and yet another guiding the decision of how much to give if one decides to contribute.

Additionally, since our scenario was considerably different from a typical exchange, where products are not freely available but sold for a fixed price, the essence of what it means to behave fairly in the current situation differed accordingly. Participants did not consider their decisions on the basis of a market price (as might be manifested by monetary contributions only slightly bellow supermarket levels) [45]; instead, as suggested by the large number of zero contributions, their decisions were significantly affected by knowing that zero contribution was also an option, however unfair this behavior might actually be. By including only true donators to our model, we only examined those who considered to behave fairly when making the decision. On account of this, we excluded zero donations from our sample and looked only at actual monetary contributions. When we analysed the non-zero contributions, we only found a significant effect of 3-dimensional agency stimuli on monetary contributions. In other words, main factors of agency and dimensionality did not yield a significant effect when analysed separately but *only* when combined as was the case in a condition with 3-dimensional stimulus suggesting on agency. These results do not provide support for the effects of 2-dimensional agency cues as reported by prior studies [6,7].

As previously noted [51], the effect of watching eyes seems to be sensitive to the type of behavioral response tested, as well as to the situational characteristics inherent to the experimental set-up [32,47]. Since our study was a field-experiment situated within a complex public material environment, we speculate, that its characteristics determined the type of stimulus that *would yield* a priming effect in a naturalistic setting like this one. Presumably, the 2-dimensional image of watching eyes (or any other agency cue administered in a pictorial form) is an appropriate way of presenting a stimulus for computer-based experiments, as documented by previous studies [6,35] but perhaps less so for studies conducted in naturalistic settings where printed image may appear particularly attention grabbing thus elicit a weak or opposed reactions. The meta-analysis of watching eyes studies conducted by Sparks and Barclay [32] helps to elucidate this issue. Strikingly, all twenty-five studies utilized 2-dimensional stimuli. Sparks and Barclay's meta-analysis suggests that length of exposure to an agency stimulus is a critical variable influencing its effects. One possible explanation for this decrease in responsiveness according to exposure time is habituation. Additionally, prolonged exposure to an agency stimulus increases the chances that a person will recognize such a stimulus to be a false cue of actual human agency [32]. Exposure time to the agency stimuli in the present study was relatively uncontrolled, meaning that habituation or scrutiny of stimuli may have taken place, at least with a subset of the sample. This possibly indicates that 2-dimensional experimental cues were disclosed as fake signals [32] and therefore were unable to evoke the prosocial effect especially since such effects seem to be driven by unconscious influences and mechanisms [33]. Our 3D agency cue may appear more appropriate within a naturalistic environment and it may be less sensitive to such habituation or false cue detection and thus more likely to produce a prosocial effect in a naturalistic setting. This assumption is also consistent with the paradigm of material priming [52] that builds upon, yet extends the long tradition of psychological studies of priming (and, particularly, semantic priming) [9,53] but more specifically investigates the priming effects of real-world objects and artifacts. The effect of material primes is documented in such domains as perceptual biases [54,55]; activation of behavioral representation [56], and behavioural goal activation [57–59].

We further propose that the effect of 3-dimensional prosocial primes in naturalistic settings may indeed be related to their spatial and “embodied” qualities. Such stimuli serve as symbolic means that represent the target domain, activating concepts or behaviors associated with this domain. Dimensionality allows the experimental stimuli to fit well within the public niche where our study took place and also be perceived without conscious awareness (a critical factor establishing the effect of all priming studies). The object’s sensori-motor profile, that is, the way its appearance changes as you move with respect to it [60], may be a key ecological characteristic inherent exclusively to 3-dimensional objects that naturalizes the object for the perceiver by means of employing the kinds of cognitive capacities used when exposed to actual “watching eyes.”

One possible limitation of our study may lie in group purchases, that is, the potential influences on decisions made in the presence of one’s peers. However, we do not think this would have affected our study in any systematic way. Yet another potential confound may lie in the presentation of the 2-dimensional stimuli as posters on a notice board. Though the motivation for this was driven by our aim to make the stimuli as subtle as possible, in order to avoid eliciting participants’ awareness of the experimental nature of a study, it may have resulted in the weak effect observed with the 2D agency stimuli. Stimulus saliency should be carefully assessed in any subsequent studies. Additional research is needed on these factors and their potential effects. However, as it would involve substantial interventions in controlled settings, such research would inevitably move away from the naturalistic framework we maintained in this study.

By demonstrating that the spatial features of agency stimuli alter the decision-making in a prosocial task, we hope to open up a new line of studies within an existing paradigm. Future research may focus on different aspects of the stimuli which may alter their salience (e.g. size and color) or investigate characteristics like perceived animacy. Furthermore, little is known about the combined effects of complex environments with more subtle cues, such as those examined in our study. Addressing these sorts of interactions through well-designed, naturalistic studies may contribute to a deeper understanding of how human cognition is influenced by the environments in which we dwell.

## Supporting Information

S1 Appendix Experimental stimuli. Image depicts Agentive-stimuli (appearing in the study either as a 3-dimensional object or 2-dimensional print) and Non-Agentive stimuli (appearing in the study either as a 3-dimensional object or 2-dimensional print).
(PDF)

S2 Appendix Experimental set-up. This image shows experimental components, namely, experimental stimulus, study instructions, refrigerator with offered beverages, and envelopes designated for monetary contributions and a particular spatial lay-out of these elements.
(JPG)

S1 Dataset Complete experimental dataset. This SPSS data-file includes all experimental data comprising zero contribution.
(SAV)
