# Supplementary material for: It Depends Who Is Watching You: 3-D Agent Cues Increase Fairness
Source: PLoS One. 2016 Feb 9;11(2):e0148845. doi: 10.1371/journal.pone.0148845 (PMC4747577; doi:10.1371/journal.pone.0148845)
Supplement: S1 Appendix — Image depicts Agentive-stimuli (appearing in the study either as a 3-dimensional object or 2-dimensional print) and Non-Agentive stimuli (appearing in the study either as a 3-dimensional object or 2-dimensional print). (PDF) [file pone.0148845.s001.pdf]

Appendix

Experimental stimuli

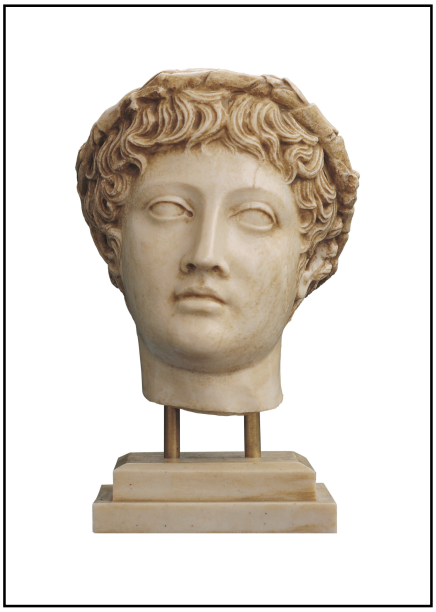

Agentive-stimuli (either as a 3-dimensional object or 2-dimensional print)

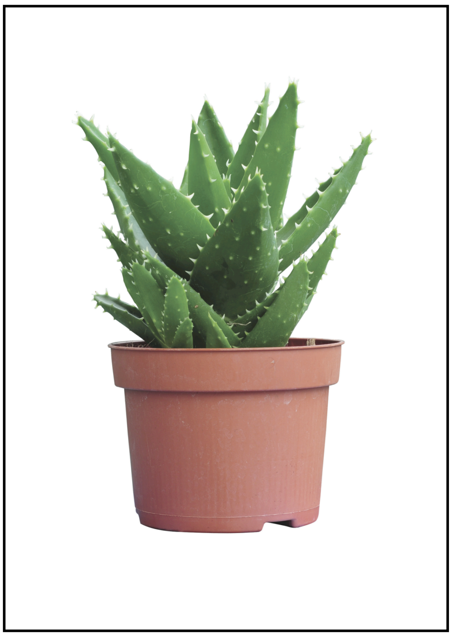

Non-Agentive stimuli (either as a 3-dimensional object or 2-dimensional print)
