# Supplementary figures and images for: It Depends Who Is Watching You: 3-D Agent Cues Increase Fairness
Source: PLoS One. 2016 Feb 9;11(2):e0148845. doi: 10.1371/journal.pone.0148845 (PMC4747577; doi:10.1371/journal.pone.0148845)

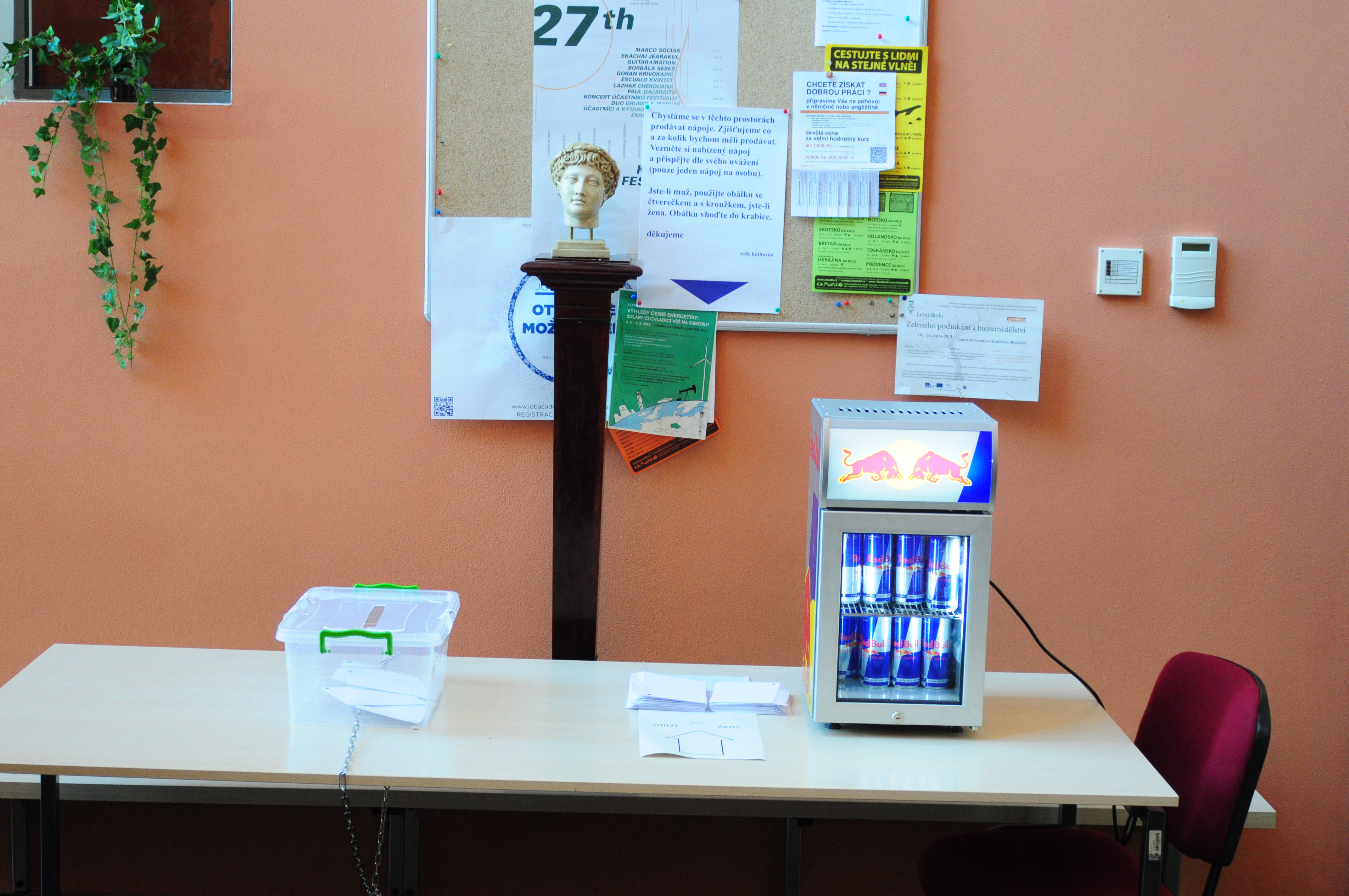

Supplement: S2 Appendix — This image shows experimental components, namely, experimental stimulus, study instructions, refrigerator with offered beverages, and envelopes designated for monetary contributions and a particular spatial lay-out of these elements. (JPG) [file pone.0148845.s002.JPG]
